# Supplementary material for: Analysis of Zika virus capsid-Aedes aegypti mosquito interactome reveals pro-viral host factors critical for establishing infection
Source: Nat Commun. 2021 May 13;12:2766. doi: 10.1038/s41467-021-22966-8 (PMC8119459; doi:10.1038/s41467-021-22966-8)
Supplement: Supplementary file 1 — Supplementary Information [file 41467_2021_22966_MOESM1_ESM.pdf]

Supplementary Information

Analysis of Zika virus capsid-*Aedes aegypti* mosquito interactome reveals pro-viral host factors critical for establishing infection

Rommel J. Gestuveo[1,2]*, Jamie Royle[1], Claire L. Donald[1#], Douglas J. Lamont[3], Edward C. Hutchinson[1], Andres Merits[4], Alain Kohl[1,5]*, and Margus Varjak[1,4]*

[1]MRC-University of Glasgow Centre for Virus Research, Glasgow G61 1QH, UK

[2]Division of Biological Sciences, University of the Philippines Visayas, Miagao, Iloilo 5023, Philippines

[3]FingerPrints Proteomics Facility, School of Life Sciences, University of Dundee, Dundee DD1 5EH, UK

[4]Institute of Technology, University of Tartu, 50411 Tartu, Estonia

[5]Lead Contact

[#]Present address: Institute of Molecular, Cell and Systems Biology, University of Glasgow, Glasgow G12 8QQ, Scotland, UK

*Correspondence: rjgestuveo@up.edu.ph (RJG), margus.varjak@glasgow.ac.uk (MV), alain.kohl@glasgow.ac.uk (AK)

Supplementary Figures 1-2

Supplementary Tables 1-6

Supplementary Reference

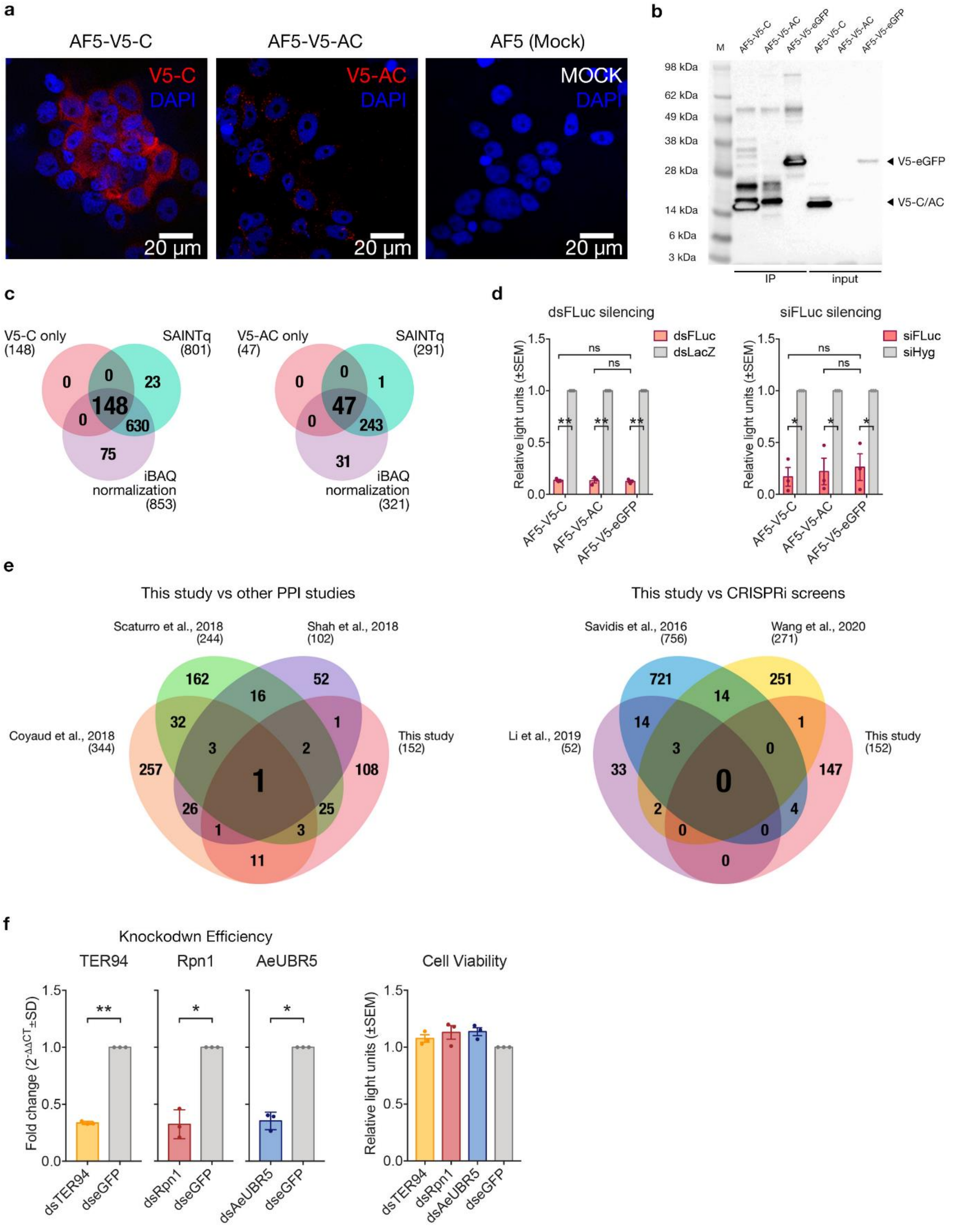

Supplementary Fig. 1. Characterization and immunoprecipitation of stable *Ae. aegypti* cell lines for proteomics and PPI analyses. **a**, Representative immunostaining of AF5-V5-AC, AF5-V5-C, and AF5 (mock) cell lines with anti-V5 antibody and DAPI (scale bar=20 µm) of n=3 independent repeats. **b**, Representative immunoblot of n=3 independent IP samples for LFQ proteomics anti-V5 antibody. **c**, Overlap of potential interactors to ZIKV C and AC between proteomics data analysis methods utilized in the study. Results of analyses provided as Supplemental Data. **d**, RNAi sensor assay previously described[1] was performed in stable AF5 mosquito cell lines. RNAi activity is measured by co-transfecting pIZ-Fluc and pAcIE1-Rluc (as transfection control) with dsRNA or siRNA

against FFLuc, allowing detection of any interference by C/AC with dsRNA cleavage or siRNA loading into silencing complexes. Data of n=3 independent experiments presented as mean±SEM light units relative to siHyg or dsLacZ controls set to 1. ns, not significant, *p-value<0.05, **p-value<0.01 determined by two-tailed Student's t-test where AF5-V5-C dsFLuc vs. dsLacZ p<0.001, siFLuc vs. siHyg p=0.011; AF5-V5-AC dsFLuc vs. dsLacZ p<0.001, siFLuc vs. siHyg p=0.026; AF5-V5-eGFP dsFLuc vs. dsLacZ p<0.001, siFLuc vs. siHyg p=0.029; AF5-V5-C vs. AF5-V5-eGFP dsFLuc p=0.564, siFLuc p=0.589; AF5-V5-AC vs. AF5-V5-eGFP dsFLuc p=0.832, siFLuc p=0.829. Exact p-values cannot be computed for p<0.001. **e**, Overlap between the 152 human orthologs of the potential mosquito interactors to ZIKV C and AC identified in this study with previous studies on ZIKV-human PPI[2–4] and genome-wide CRISPR screens[5–7] in human cells. **f**, Left panel, Efficiency of dsRNA knockdown of TER94, Rpn1, and AeUBR5 in AF5 cells was assessed by RT-qPCR showing RNA fold change ($2^{-\Delta\Delta CT}$) as mean±SD relative to S7 internal control gene and dseGFP controls set to 1 from n=3 independent replicates. *p-value<0.05, **p-value<0.01 determined by two-tailed Student's t-test where dsTER94 p<0.001 (exact p-value cannot be computed); dsRpn1 p=0.036; dsAeUBR5 p=0.016. Right panel, AF5 cell viability measured using CellTiter-Glo assay (Promega) at 24 hpt of dsTER94, dsRpn1, and dsAeUBR5 presented as relative light units (mean±SEM) to dseGFP controls set to 1 from n=3 independent replicates with p-value determined by two-tailed Student's t-test where dsTER94 p=0.151; dsRpn1 p=0.158; dsAeUBR5 p=0.065. Source Data file provided.

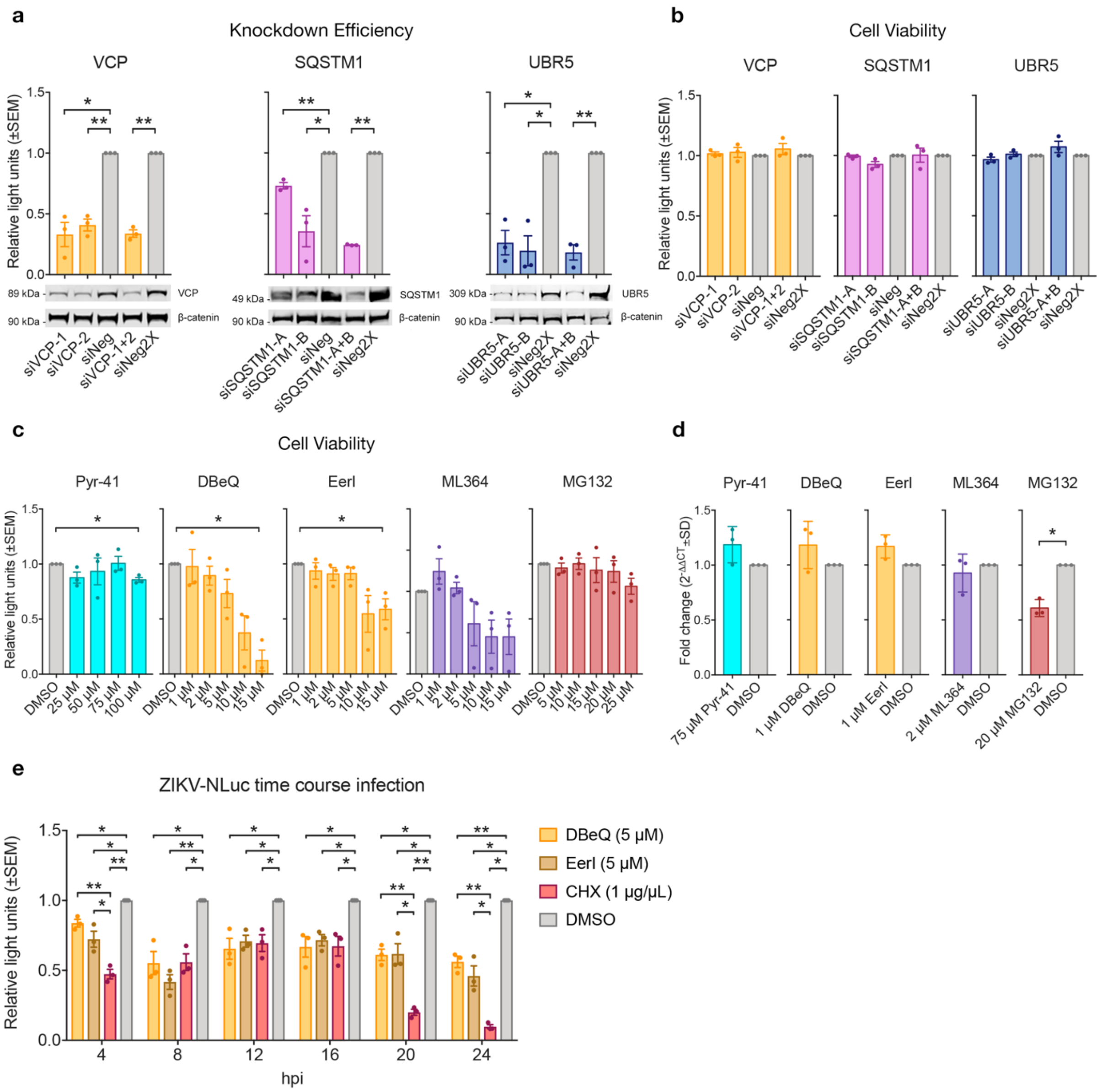

Supplementary Fig. 2. Knockdown or inhibition of proteins of interest in A549 cells during ZIKV infection. **a**, Densitometry of n=3 independent immunoblots of VCP, SQSTM1, and UBR5 siRNA knockdown in A549 cells at 72 hpt. Band intensities (β-catenin loading control) shown as mean±SEM signal relative to siNeg controls set to 1. *p-value<0.05, **p-value<0.01 determined by two-tailed Student's t-test where siVCP-1 p=0.022; siVCP-2 p=0.007; siVCP-1+2 p=0.002; siSQSTM1-A p=0.009; siSQSTM1-B p=0.037; siSQSTM1-A+B p<0.01; siUBR5-A p=0.018; siUBR5-B p=0.023; siUBR5-A+B p=0.006. Representative immunoblots shown with anti-VCP, anti-SQSTM1, anti-UBR5 and anti-β-catenin antibodies. **b**, Cell viability of A549 cells at 72 hpt of individual siRNAs, or in combination, against VCP, SQTSM1, and UBR5 using CellTiter-Glo assay (Promega) with luciferase readings from n=3 independent replicates shown as mean±SEM light units relative to siNeg controls set to 1 with p-value determined by two-tailed Student's t-test where siVCP-1 p=0.409; siVCP-2 p=0.573; siVCP-1+2 p=0.358; siSQSTM1-A p=0.600; siSQSTM1-B p=0.096; siSQSTM1-A+B p=962; siUBR5-A p=0.596; siUBR5-B p=0.619; siUBR5-A+B p=0.264. **c**, Cell viability of A549 cells treated with chemical inhibitors at different concentrations for 24 h and measured using CellTiter-Glo assay (Promega) with luciferase readings from n=3 independent replicates shown as mean±SEM light units relative to DMSO controls set to 1. *p-value<0.05, **p-value<0.01 determined by two-tailed Student's t-test where Pyr-41 25 μM p=0.132, 50 μM p=0.638, 75 μM p=0.928, 100 μM p=0.023; DBeQ 1 μM p=0.891, 2 μM p=0.344, 5 μM p=0.171, 10 μM p=0.056, 15 μM p=0.011; EerI 1 μM p=0.481, 2 μM p=0.255, 5 μM p=0.261, 10 μM p=0.113, 15 μM p=0.049; ML364 1 μM p=0.257, 2 μM p=0.602, 5 μM p=0.281 10 μM p=0.112, 15 μM p=0.125; MG132 5 μM p=0.493, 10 μM p=0.975, 15 μM p=0.666, 20

μM p=0.557, 25 μM p=0.108. **d**, A549 cells treated with chemical inhibitors 2 hpi of ZIKV PE243 (MOI=10) with n=3 independent experiments presented as mean fold change ($2^{-\Delta\Delta CT}$±SD) of CT values normalized to GAPDH and DMSO controls set to 1. *p-value<0.05 determined by two-tailed Student's t-test where Pyr-41 p=0.189; DBeQ p=0.300; EerI p=102; ML364 p=0.345; MG132 p=0.019. **e**, ZIKV time course infection in A549 cells treated with DBeQ, EerI, or cycloheximide (CHX) at 2 h prior to infection with ZIKV-NLuc (MOI=10). NLuc readings of n=3 independent repeats measured at 4 h intervals and presented as mean±SEM light units relative to DMSO controls set to 1. *p-value<0.05 and **p-value<0.01 determined by two-tailed Student's t-test where at 4 hpi vs. DMSO DBeQ p=0.028, EerI p=0.039, CHX p=0.004; at 8 hpi vs. DMSO DBeQ p=0.032, EerI p=0.008, CHX p=0.018; 12 hpi vs. DMSO DBeQ p=0.043, EerI p=0.020, CHX p=0.036; 16 hpi vs. DMSO DBeQ p=0.046, EerI p=0.019, CHX p=0.043; 20 hpi vs. DMSO DBeQ p=0.011, EerI p=0.035, CHX p<0.001; 24 hpi vs. DMSO DBeQ p=0.009, EerI p=0.017, CHX p<0.001; 4 hpi vs. CHX DBeQ p=0.001, EerI p=0.028; 8 hpi vs. CHX DBeQ p=0.953, EerI p=0.148; 12 hpi vs. CHX DBeQ p=0.697, EerI p=0.861; 16 hpi vs. CHX DBeQ p=0.965, EerI p=0.629; 20 hpi vs. CHX DBeQ p=0.003, EerI p=0.023; 24 hpi vs. CHX DBeQ p=0.003, EerI p=0.033. Source Data file provided.

Supplementary Table 1. *Ae. aegypti* protein interactors of ZIKV C.

No.	UniProt ID	VectorBase ID	Dmel ortholog	Hsap ortholog	No. proteins	No. peptides	MaxQuant Score	Q-value	iBAQ_C1	iBAQ_C2	iBAQ_C3	Mean iBAQ	LFQ_C1	LFQ_C2	LFQ_C3	Mean LFQ
1160	Q16EQ1	AAEL015065	FBgn0250789	SPTAN1	4	141	323.31	0	8065.1	8997200	1537300	3514188.37	300840	192820000	63515000	85545280
1154	A0A6I8TH48	AAEL009070	FBgn0261618	LARP1B	2	56	323.31	0	120190	18028000	18447000	12198396.7	6254800	207490000	373220000	195654933
1153	A0A6I8TT17	AAEL019420	FBgn0052654	SEC16A	6	54	323.31	0	9031.4	610190	2878600	1165940.47	589980	8531800	64697000	24606260
1151	A0A6I8T7Y3	AAEL003415	FBgn0002525	LMNA	2	46	323.31	0	2715600	6603100	1924000	3747566.67	73725000	40057000	20682000	44821333.3
1146	Q16JS2	AAEL013233	FBgn0000146	PIWIL1	3	41	323.31	0	29044	4021400	3067200	2372548	1224500	34878000	46536000	27546166.7
1144	A0A1S4FYI5	AAEL013098	FBgn0025725	COPA	2	40	323.31	0	32112	1535700	1357900	975237.333	1286500	18578000	31022000	16962166.7
1142	A0A6I8T3F0	AAEL000276	FBgn0034641	DCAF1	5	37	323.31	0	38212	225170	748880	337420.667	2870300	4408900	18850000	8709733.33
1139	A0A6I8TIN5	AAEL010146	FBgn0028479	HADHA	1	36	323.31	0	79330	2009300	4937500	2342043.33	3075700	14671000	62866000	26870900
1141	Q16IV9	AAEL013530	FBgn0015509	CUL1	1	36	323.31	0	20804	1669000	1606200	1098668	1283000	7731000	24388000	11134000
1136	Q16NZ4	AAEL011815	FBgn0010247	PARP1	1	34	323.31	0	827670	179330	20224	342408	35788000	1848800	338770	12658523.3
1134	A0A6I8T8D6	AAEL005861	FBgn0261260	LRP2	1	33	323.31	0	25882	250370	125050	133767.333	2751400	4958300	4802900	4170866.67
1128	A0A1S4EZS3	AAEL001769	FBgn0035720	DDX17	1	32	323.31	0	163240	6474800	3715500	3451180	6362900	44355000	41939000	30885633.3
1127	A0A6I8TNG8	AAEL012171	FBgn0027548	RBM15B	6	32	323.31	0	32943	4177700	4020000	2743547.67	1191200	26967000	43286000	23814733.3
1129	Q17CI5	AAEL004546	FBgn0008635	COPB1	1	32	323.31	0	81453	1556700	1078000	905384.333	2641800	11236000	15413000	9763600
1126	A0A6I8U795	AAEL025927	FBgn0030918	FBXL13	1	31	323.31	0	82111	8952700	4594200	4543003.67	1579500	42975000	42739000	29097833.3
1123	Q16Y63	AAEL008658	FBgn0036587	IGFALS	1	30	323.31	0	324660	6077100	3185200	3195653.33	9890800	35504000	31638000	25677600
1122	Q16TN4	AAEL010159	FBgn0087013	IPO5	1	30	323.31	0	10454	1047700	349740	469298	849740	9429400	5661200	5313446.67
1124	Q174T8	AAEL006794	FBgn0034246	DICER1	1	30	323.31	0	14683	354490	252020	207064.333	1290700	4599300	6553700	4147900
1117	A0A6I8U7S6	AAEL024841	FBgn0025724	COPB2	2	29	323.31	0	17039	1283000	1066200	788746.333	2190300	6738400	10753000	6560566.67
1112	A0A6I8TEL1	AAEL006876	FBgn0285926	IGF2BP1	1	27	323.31	0	103930	14804000	19106000	11337976.7	1921300	71987000	162770000	78892766.7
1113	A0A6I8U583	AAEL027249	FBgn0028968	COPG1	1	27	323.31	0	69569	1690300	1338900	1032923	2352400	12278000	18720000	11116800
1107	Q174Z2	AAEL006751	FBgn0027544	-	1	26	323.31	0	873730	4706400	4963400	3514510	29175000	32431000	54779000	38795000
1098	M9NCK7	AAEL021134	FBgn0263391	ADD1	4	26	323.31	0	159970	7815700	2551400	3509023.33	5675400	36901000	18389000	20321800
1100	A0A1S4F477	AAEL003293	FBgn0034243	GNL2	1	26	323.31	0	38107	2493000	1021200	1184102.33	886500	15560000	8421600	8289366.67
1105	A0A6I8U3U1	AAEL022113	FBgn0005666	TTN	1	26	323.31	0	73477	551860	582640	402659	4965400	5952400	10347000	7088266.67
1106	A0A6I8U6N0	AAEL023850	FBgn0005674	EPRS	1	26	323.31	0	88714	114390	37652	80252	6672900	2021800	1868600	3521100
1092	A0A6I8TFU5	AAEL008171	FBgn0263603	ZFR	2	25	323.31	0	40945	1872700	835190	916278.333	1757300	11587000	10918000	8087433.33
1093	A0A6I8U1U7	AAEL022819	FBgn0000319	CLTC	2	25	228.63	0	73694	257200	44353	125082.333	5725700	3615100	2085100	3808633.33
1089	A0A1S4F1P1	AAEL002407	FBgn0260962	DDB1	1	24	252.58	0	81368	604590	896940	527632.667	3737200	4676000	11190000	6534400
1087	Q17EV4	AAEL003670	FBgn0039335	VPS33B	1	23	323.31	0	178930	11399000	8997400	6858443.33	3069400	29144000	57624000	29945800
1085	Q16LC6	AAEL012690	FBgn0037901	EXD2	1	23	323.31	0	16726	2046200	2927400	1663442	1104800	20947000	19775000	13942266.7
1072	A0A6I8T510	AAEL002178	FBgn0263594	MTHFSD	3	22	323.31	0	232750	6575800	5582800	4130450	3987400	23864000	38796000	22215800
1071	A0A1S4FCM2	AAEL006095	FBgn0010225	GSN	3	22	323.31	0	131960	3254800	1254200	1546986.67	3548100	19383000	13472000	12134366.7
1073	Q17B45	AAEL005052	FBgn0003890	TUBB2A	2	22	144.57	0	109670	1479300	258960	615976.667	1490600	3931800	1782900	2401766.67
1080	Q16SH1	AAEL010585	FBgn0286784	VCP	1	22	323.31	0	232450	296530	186990	238656.667	7943600	2352800	3324300	4540233.33
1063	Q0IG45	AAEL003514	FBgn0259937	DKC1	2	21	268.51	0	62202	5412600	1992700	2489167.33	1697200	23296000	17080000	14024400
1064	A0A6I8TD93	AAEL006582	FBgn0263006	ATP2A1	2	21	285.5	0	167410	1068600	637810	624606.667	5820400	5113700	7796000	6243366.67
1062	A0A6I8TQ85	AAEL014931	FBgn0262579	SARM1	7	21	257.59	0	33650	429070	235390	232703.333	2345600	3782800	3934200	3354200

1051	A0A1S4FKA5	AAEL008687	FBgn0032515	PRKRA	2	20	323.31	0	48833	4654600	2764200	2489211	723170	14422000	18489000	11211390
1050	Q16YK5	AAEL008517	FBgn0024556	TUFM	3	20	243.39	0	806010	2161200	2100300	1689170	15883000	10486000	14656000	13675000
1060	Q16UJ3	AAEL009883	FBgn0287225	ATAD3A	1	20	191.54	0	129970	1200200	1970300	1100156.67	5055300	7267500	19608000	10643600
1049	A0A6I8TDU5	AAEL006362	FBgn0028646	SLC25A12	4	20	311.16	0	99275	1640000	1229600	989625	3086100	7395900	11125000	7202333.33
1059	Q16EU8	AAEL020382	FBgn0012034	ACSS2	1	20	323.31	0	342850	957930	1090800	797193.333	9824500	5121700	10961000	8635733.33
1056	A0A1S4FSP5	AAEL011282	FBgn0033741	NOP2	1	20	271.03	0	140330	1348800	473940	654356.667	5201000	6508100	5040000	5583033.33
1048	Q0IFN2	AAEL004500	FBgn0000559	EEF2	2	20	219.77	0	234350	873050	111410	406270	7726200	4401500	2160200	4762633.33
1055	A0A1S4FM94	AAEL009416	FBgn0038016	PHF20L1	1	20	227.77	0	12476	543840	620690	392335.333	817940	4105200	11227000	5383380
1057	A0A1S4FW12	AAEL012243	FBgn0004227	SFPQ	1	20	200.61	0	42790	983630	109130	378516.667	1377300	6842300	1829900	3349833.33
1042	Q16SW8	AAEL010467	FBgn0001215	HNRNPA2B1	1	19	219.22	0	65600	7553700	6761800	4793700	1305800	13960000	37853000	17706266.7
1032	Q16VV7	AAEL009422	FBgn0004401	CIZ1	8	19	309.51	0	199360	3993000	2029400	2073920	5002300	19349000	20032000	14794433.3
1036	Q16RY3	AAEL010787	FBgn0003261	DDX17	2	19	323.31	0	31452	2254500	553950	946634	800890	10592000	5910600	5767830
1031	A0A6I8TQC8	AAEL023148	FBgn0014141	FLNA	9	19	213.15	0	40564	46616	29034	38738	4184100	1362700	1320500	2289100
1023	A0A1S4G5W3	AAEL017116	FBgn0004227	SFPQ	3	18	323.31	0	705620	6477400	1529300	2904106.67	14746000	32844000	21057000	22882333.3
1024	A0A6I8T7V1	AAEL005277	FBgn0027567	CIZ1	2	18	276.67	0	65626	1797800	1389700	1084375.33	1925700	9753800	14216000	8631833.33
1028	Q16QL1	AAEL011255	FBgn0038473	GNL3L	1	18	205.9	0	33272	597980	80237	237163	1259400	2652600	1032400	1648133.33
1020	A0A6I8U0E2	AAEL021795	-	-	1	17	182.42	0	517740	24462000	6277000	10418913.3	10128000	61377000	37102000	36202333.3
1021	A0A6I8U913	AAEL028182	FBgn0035880	LRP3	1	17	288.44	0	117900	1575500	618910	770770	1878200	6952300	4916400	4582300
1009	A0A6I8TVN6	AAEL019669	FBgn0022959	YBX1	1	16	323.31	0	161340	32790000	42645000	25198780	2300300	84683000	155470000	80817766.7
1006	A0A1S4F5J5	AAEL003726	FBgn0028427	ILK	1	16	323.31	0	52897	2876000	859460	1262785.67	1682800	14466000	7808000	7985600
1012	Q176M6	AAEL006315	FBgn0028695	PSMD2	1	16	163.99	0	64375	255100	160740	160071.667	2552100	1776300	2335300	2221233.33
987	Q174U3	AAEL006785	FBgn0010409	RPL18A	2	15	196.24	0	2790300	41814000	15184000	19929433.3	21272000	51984000	28268000	33841333.3
986	Q1HR74	AAEL024536	FBgn0285950	RPL19	2	15	207.65	0	1572700	47777000	8354000	19234566.7	10979000	50620000	20184000	27261000
994	Q16HL9	AAEL013982	FBgn0011640	RBM4	1	15	277.99	0	162450	19541000	12207000	10636816.7	3166300	67764000	91821000	54250433.3
996	Q176A9	AAEL006473	FBgn0283477	SRSF1	1	15	114.68	0	181340	8476700	7650700	5436246.67	2945000	18474000	22652000	14690333.3
993	J9EAN1	AAEL017421	FBgn0038964	NOP56	1	15	323.31	0	155430	4429900	2180400	2255243.33	2965900	15713000	15543000	11407300
992	A0A6I8U4P3	AAEL026751	FBgn0003231	SQSTM1	1	15	243.12	0	81912	1539200	1757600	1126237.33	1860100	5288800	11125000	6091300
988	A0A6I8T3U9	AAEL001009	FBgn0027598	SH3KBP1	1	15	181.85	0	670060	481630	320000	490563.333	17921000	1789600	4217800	7976133.33
980	A0A6I8U015	AAEL022091	FBgn0286898	MIA2	3	15	310.48	0	10378	78930	158070	82459.3333	443920	635660	2232200	1103926.67
979	A0A6I8TTL3	AAEL018168	FBgn0037027	CDY1	4	15	146.7	0	31729	93413	64003	63048.3333	2247600	1061100	1039700	1449466.67
978	Q1HRV1	AAEL010168	FBgn0004867	RPS2	1	14	280.64	0	992320	32273000	10459000	14574773.3	20080000	78134000	30207000	42807000
964	Q17H73	AAEL002761	FBgn0003721	TPM1	12	14	249.6	0	53882	5056100	753400	1954460.67	1098600	15782000	2517000	6465866.67
974	Q17IM1	AAEL002296	FBgn0025352	HADHB	1	14	175.22	0	22241	1388000	3488700	1632980.33	545510	5438500	25467000	10483670
968	A0A1S4FE42	AAEL006634	FBgn0029969	ACAT1	1	14	166.39	0	529220	1253200	1307500	1029973.33	8284400	3771000	7552300	6535900
956	A0A6I8TPB5	AAEL013888	FBgn0039600	PRPF39	2	14	121.52	0	15075	167640	213230	131981.667	864710	1737600	3108900	1903736.67
957	A0A6I8T2V8	AAEL000081	FBgn0016983	NVL	2	14	137.18	0	57044	145920	58670	87211.3333	2912800	1397600	1513900	1941433.33
951	Q16ZH3	AAEL008188	FBgn0039857	RPL6	1	13	150.07	0	2629700	50515000	16998000	23380900	18923000	63748000	40997000	41222666.7
944	A0A6I8TWT1	AAEL019778	FBgn0050122	HNRNPUL1	1	13	323.31	0	1668700	25618000	22602000	16629566.7	13218000	40795000	69155000	41056000
952	Q173Y9	AAEL006977	FBgn0260439	PPP2R1A	1	13	100.41	0	77558	616130	268540	320742.667	2394400	2820100	3216700	2810400
954	Q17ME6	AAEL001037	FBgn0030720	FTSJ3	1	13	192.5	0	62895	161330	50273	91499.3333	1597200	1212000	643280	1150826.67
909	Q172D2	AAEL007439	FBgn0004687	MYL6	2	12	283.35	0	495940	40370000	7869500	16245146.7	3197400	52440000	19433000	25023466.7

896	Q1HR34	AAEL003396	FBgn0002626	RPL32	2	12	198.45	0	2150600	28557000	9365600	13357733.3	9750400	31316000	14293000	18453133.3
928	Q17EQ5	AAEL003664	FBgn0011638	SSB	1	12	118.43	0	99372	1911000	1216100	1075490.67	1825600	7096400	7457900	5459966.67
921	J9EB97	AAEL017508	FBgn0027610	SLC25A10	1	12	99.527	0	113920	1737700	1066400	972673.333	1676700	3956200	4210300	3281066.67
897	A0A6I8TDE9	AAEL006684	FBgn0043456	GLYR1	11	12	152.29	0	167350	782780	492140	480756.667	3853400	2547400	3120700	3173833.33
902	A0A6I8TLY7	AAEL010991	FBgn0263120	ACSL3	3	12	80.126	0	36985	141490	507180	228551.667	1143700	1153100	4913300	2403366.67
917	A0A1S4FNZ2	AAEL009859	FBgn0028473	GTPBP4	1	12	91.756	0	32046	342120	238590	204252	1175900	2592400	2373300	2047200
905	Q0IE87	AAEL013275	FBgn0262743	KPNB1	2	12	302.29	0	15991	383730	201350	200357	634940	2125800	2144400	1635046.67
922	Q16P69	AAEL011745	FBgn0036915	PRPF3	1	12	86.512	0	24260	368130	194050	195480	797050	1931800	2155400	1628083.33
930	Q17H99	AAEL002748	FBgn0002069	DARS	1	12	213.46	0	103160	169890	73448	115499.333	3241400	1272000	1493100	2002166.67
848	Q9GSB1	AAEL002372	FBgn0033699	RPS11	2	11	169.93	0	2345200	66568000	17500000	28804400	18247000	89744000	46864000	51618333.3
894	Q1HRT6	AAEL002534	FBgn0024733	RPL10L	1	11	98.881	0	1510500	70758000	10162000	27476833.3	14527000	66041000	33038000	37868666.7
892	Q1HR32	AAEL000987	FBgn0261602	RPL8	1	11	241.14	0	1281100	44086000	9327100	18231400	12449000	63970000	30013000	35477333.3
891	Q17PV7	AAEL000210	FBgn0036135	MRPL2	1	11	88.3	0	79803	2421400	1078500	1193234.33	2154000	4523300	6752800	4476700
859	A0A6I8TJC5	AAEL010576	FBgn0002781	BTBD18	46	11	208.52	0	254640	403930	367270	341946.667	3578900	1549500	2398500	2508966.67
863	Q0IFL2	AAEL004686	FBgn0040078	RUVBL1	1	11	208.4	0	110560	342380	211150	221363.333	2576500	1177100	1366800	1706800
876	Q16EF6	AAEL015216	FBgn0027889	VRK1	1	11	160.01	0	307920	173910	35080	172303.333	6527300	1029500	499710	2685503.33
855	Q8WRS8	AAEL023255	FBgn0011703	RRM1	2	11	79.256	0	75742	242480	27065	115095.667	1494200	2165900	955100	1538400
890	Q17NS6	AAEL000622	FBgn0037855	TTC14	1	11	119.36	0	82807	84942	167420	111723	2688600	913210	1569900	1723903.33
811	Q1HRM9	AAEL014583	FBgn0003274	RPLP2	2	10	206.32	0	4148100	103690000	21206000	43014700	13784000	86189000	25456000	41809666.7
836	Q16KZ2	AAEL012827	FBgn0039562	HSP90B1	1	10	88.386	0	125240	127880	97384	116834.667	3410100	1151800	1469200	2010366.67
821	A0A6I8TQ25	AAEL014702	FBgn0027084	KARS	2	10	175.51	0	30827	240540	61135	110834	861230	1117800	795550	924860
826	A0A1S4EV92	AAEL000199	FBgn0027093	RARS	1	10	89.299	0	45087	102410	116230	87909	1526800	970780	1098000	1198526.67
842	Q17HZ7	AAEL002508	FBgn0028684	PSMC3	1	10	141.89	0	37298	153840	42665	77934.3333	793100	649620	737280	726666.667
805	Q1HR35	AAEL005220	FBgn0086710	RPL30	1	9	147.56	0	2887100	64901000	15877000	27888366.7	14148000	57321000	28934000	33467666.7
808	Q1HRP0	AAEL020749	FBgn0285948	RPL27A	1	9	56.901	0	632090	34104000	7460100	14065396.7	8865900	30064000	17520000	18816633.3
772	Q16YF3	AAEL008562	FBgn0033581	PLAG1	2	9	86.956	0	109530	2271000	2136200	1505576.67	3310200	7421100	9893200	6874833.33
788	A0A1S4F2V5	AAEL002833	FBgn0013770	CTSV	1	9	203.15	0	111850	2381800	1379000	1290883.33	1873000	5811900	6061800	4582233.33
790	A0A1S4F7M2	AAEL004338	FBgn0039635	PDHB	1	9	88.751	0	260990	385340	256190	300840	4428300	1353100	2164300	2648566.67
771	Q16YE9	AAEL008565	FBgn0036702	AFG3L2	2	9	79.796	0	19233	85609	142480	82440.6667	951920	615830	1298000	955250
789	A0A1S4F345	AAEL002906	FBgn0028692	PSMD1	1	9	93.767	0	31429	137430	55105	74654.6667	1376900	952000	1136100	1155000
786	A0A1S4EWJ6	AAEL000682	FBgn0032717	RSBN1	1	9	68.077	0	24179	155330	26839	68782.6667	1262500	1139000	868600	1090033.33
718	Q1HRP7	AAEL003530	FBgn0002593	RPLP1	5	8	110.72	0	9976000	160880000	43220000	71358666.7	29768000	108700000	51966000	63478000
767	Q52UT2	AAEL004175	FBgn0005533	RPS17	1	8	168.3	0	2210100	77354000	3268400	27610833.3	10361000	53479000	7225700	23688566.7
766	Q1HRQ6	AAEL009151	FBgn0010198	RPS15A	1	8	94.301	0	982560	42592000	2786400	15453653.3	7463900	56792000	4770300	23008733.3
754	Q178R6	AAEL005817	FBgn0036825	RPL26	1	8	255.68	0	849380	35989000	9300900	15379760	4659800	27878000	13511000	15349600
710	Q179T1	AAEL005515	FBgn0263396	HNRNPAB	6	8	323.31	0	655580	3791500	2515700	2320926.67	5580400	7957900	8216200	7251500
759	Q17GJ0	AAEL003001	FBgn0040066	WDR5	1	8	89.534	0	56259	1564300	1249000	956519.667	1133300	3322000	4768600	3074633.33
751	Q170U8	AAEL007803	FBgn0034657	LBR	1	8	103.36	0	84086	279890	237060	200345.333	1716100	1344100	1465100	1508433.33
732	Q16TA2	AAEL010341	FBgn0040075	RUVBL2	1	8	54.808	0	178340	165570	128710	157540	3420600	1159200	1685600	2088466.67
703	Q17B41	AAEL005097	FBgn0011272	RPL13	1	7	92.106	0	3044300	62164000	11704000	25637433.3	14850000	61510000	24748000	33702666.7
697	Q16PF7	AAEL011656	FBgn0034138	RPS15	1	7	106.13	0	859010	28236000	6487300	11860770	4110000	39583000	10369000	18020666.7

690	Q0IG01	AAEL003750	FBgn0016685	NPM3	1	7	217.18	0	738020	8967400	1289400	3664940	6432700	9115300	2914200	6154066.67
694	Q16JE9	AAEL013353	FBgn0000308	PFN4	1	7	80.779	0	1397900	1222300	1376000	1332066.67	8140600	1584500	3007900	4244333.33
669	Q8WSF6	AAEL019408	FBgn0040309	PRDX2	2	7	58.144	0	939270	529410	150390	539690	8925700	1015400	960950	3634016.67
673	Q1HQX9	AAEL019717	FBgn0026409	SLC25A3	4	7	90.161	0	105310	1331600	126020	520976.667	1587100	3178100	1487700	2084300
707	Q1HR78	AAEL000641	FBgn0286818	P4HB	1	7	156.76	0	307370	144300	168430	206700	5191400	836210	1894100	2640570
681	A0A1S4FXA4	AAEL012826	FBgn0010173	RPA1	1	7	93.177	0	35060	354940	103520	164506.667	1261200	1397400	1100100	1252900
595	Q16FB1	AAEL011587	FBgn0039359	RPL27	2	6	48.567	0	3496800	75228000	11587000	30103933.3	12994000	54022000	17966000	28327333.3
603	Q1HR65	AAEL011471	FBgn0029897	RPL17-C18orf32	1	6	58.474	0	2458900	26492000	4650000	11200300	8805900	25413000	19166000	17794966.7
608	A0A1S4F1W0	AAEL002501	FBgn0030329	TXNDC5	1	6	125.13	0	105370	297970	194310	199216.667	1836800	880410	1161100	1292770
632	Q16PM9	AAEL011584	FBgn0015245	HSPD1	1	6	51.405	0	119360	122150	124700	122070	2591500	649030	1179600	1473376.67
578	Q1HR85	AAEL008329	FBgn0032518	RPL24	1	5	46.944	0	1056200	28054000	4972300	11360833.3	5873500	20544000	8948500	11788666.7
487	Q1DGV3	AAEL024999	FBgn0053834	H1-4	8	5	56.115	0	21531000	2752800	2550600	8944800	94030000	3022800	3999100	33683966.7
563	Q16WZ0	AAEL009041	FBgn0284255	ARPC4	1	5	44.789	0	92153	2144300	557110	931187.667	1148700	2269700	1925800	1781400
526	A0A1S4FKB1	AAEL008740	FBgn0035471	TECR	1	5	67.869	0	78103	733630	798450	536727.667	776800	1240500	1917900	1311733.33
518	A0A1S4EZB3	AAEL001623	FBgn0029133	PSME3	1	5	41.065	0	65068	767660	767080	533269.333	1373200	1676300	2709500	1919666.67
577	Q1HR40	AAEL008257	FBgn0004838	DAZAP1	1	5	137.36	0	89512	559600	242780	297297.333	905750	826080	718940	816923.333
557	Q16QZ7	AAEL011116	FBgn0020238	YWHAE	1	5	112.36	0	137650	449210	227800	271553.333	1628500	902820	1028600	1186640
574	Q17MM5	AAEL000951	FBgn0028737	EEF1B2	1	5	64.409	0	252930	176440	58523	162631	1941600	635140	827920	1134886.67
498	Q171G0	AAEL007650	FBgn0030086	CCT2	2	5	66.431	0	41575	51007	29644	40742	703830	448950	479340	544040
481	Q1HRN4	AAEL007715	FBgn0032987	RPL21	1	4	32.386	0	1238500	45407000	11729000	19458166.7	10399000	33041000	16787000	20075666.7
393	Q1HR17	AAEL000010	FBgn0002579	RPL36	2	4	28.616	0	1208800	44073000	9998300	18426700	3486900	18780000	7417400	9894766.67
467	Q17N60	AAEL000823	FBgn0037328	RPL35A	1	4	25.33	0	338630	13699000	1958100	5331910	3214600	13601000	7682200	8165933.33
406	Q1HR79	AAEL003872	FBgn0037874	TPT1	1	4	75.189	0	554780	559600	423500	512626.667	3411500	777670	863480	1684216.67
477	Q1HQY1	AAEL012095	FBgn0015282	PSMC1	1	4	35.952	0	45176	231710	93330	123405.333	1181800	613380	680480	825220
437	Q16N14	AAEL012122	FBgn0261396	PSMD3	1	4	31.983	0	31118	94740	42181	56013	948310	456300	533640	646083.333
268	Q16IV1	AAEL013536	FBgn0003942	RPS27A	1	3	86.43	0	945550	20589000	7122200	9552250	1908400	5897400	3688900	3831566.67
374	Q1HR76	AAEL013272	FBgn0261608	RPL37A	1	3	39.103	0	531710	11551000	2964000	5015570	2014600	5418300	4136300	3856400
224	Q0IEW2	AAEL007765	FBgn0033112	SERPINI1	8	3	79.703	0	65086	90006	104130	86407.3333	768910	379580	503740	550743.333

Supplementary Table 2. *Ae. aegypti* protein interactors of ZIKV AC.

No.	UniProt ID	VectorBase ID	Dmel ortholog	Hsap ortholog	No. proteins	No. peptides	MaxQuant Score	Q-value	iBAQ_AC1	iBAQ_AC2	iBAQ_AC3	Mean iBAQ	LFQ_AC1	LFQ_AC2	LFQ_AC3	Mean LFQ
487	Q1DGV3	AAEL024999	FBgn0053834	H1-4	8	5	56.115	0	100830000	239380	378200	33815860	206300000	2849500	4244000	71131166.7
1151	A0A6I8T7Y3	AAEL003415	FBgn0002525	LMNA	2	46	323.31	0	31865000	33735	173750	10690828.3	286610000	2367800	11310000	100095933
703	Q17B41	AAEL005097	FBgn0011272	RPL13	1	7	92.106	0	22211000	1811100	153800	8058633.33	47139000	19460000	3548200	23382400
690	Q0IG01	AAEL003750	FBgn0016685	NPM3	1	7	217.18	0	18041000	476770	815410	6444393.33	24585000	6000100	9281300	13288800
986	Q1HR74	AAEL024536	FBgn0285950	RPL19	2	15	207.65	0	10511000	1453400	145470	4036623.33	29911000	20980000	2324900	17738633.3
944	A0A6I8TWT1	AAEL019778	FBgn0050122	HNRNPUL1	1	13	323.31	0	9121500	212340	403240	3245693.33	25508000	4947300	7430100	12628466.7
896	Q1HR34	AAEL003396	FBgn0002626	RPL32	2	12	198.45	0	6758100	1179700	248570	2728790	20499000	13431000	3432200	12454066.7
978	Q1HRV1	AAEL010168	FBgn0004867	RPS2	1	14	280.64	0	6588200	1300300	167900	2685466.67	22890000	40468000	22875000	28744333.3
1009	A0A6I8TVN6	AAEL019669	FBgn0022959	YBX1	1	16	323.31	0	5110600	235540	139600	1828580	13576000	6778400	4507600	8287333.33
1123	Q16Y63	AAEL008658	FBgn0036587	IGFALS	1	30	323.31	0	3436300	117900	256540	1270246.67	33862000	8467600	17957000	20095533.3
1099	A0A6I8TZM0	AAEL021625	FBgn0031990	DDHD2	2	26	248.95	0	22283	1759200	1416400	1065961	160800	126780000	110140000	79026933.3
1023	A0A1S4G5W3	AAEL017116	FBgn0004227	SFPQ	3	18	323.31	0	2739800	47633	57008	948147	21047000	3908200	4611600	9855600
919	A0A6I8U337	AAEL025078	FBgn0024509	SEC13	1	12	98.174	0	1433300	251210	1108400	930970	4961500	9148200	19377000	11162233.3
1050	Q16YK5	AAEL008517	FBgn0024556	TUFM	3	20	243.39	0	2468500	140580	126180	911753.333	18904000	8916800	14269000	14029933.3
968	A0A1S4FE42	AAEL006634	FBgn0029969	ACAT1	1	14	166.39	0	1708800	87604	61525	619309.667	10243000	3964900	3102200	5770033.33
1153	A0A6I8TT17	AAEL019420	FBgn0052654	SEC16A	6	54	323.31	0	772690	62990	925100	586926.667	21180000	12256000	118760000	50732000
988	A0A6I8T3U9	AAEL001009	FBgn0027598	SH3KBP1	1	15	181.85	0	1715400	12158	22815	583457.667	19282000	926970	1595100	7268023.33
1059	Q16EU8	AAEL020382	FBgn0012034	ACSS2	1	20	323.31	0	1434600	23510	115160	524423.333	12275000	1721000	9243200	7746400
593	Q174F2	AAEL006915	FBgn0023517	PGAM5	2	6	70.502	0	878780	306760	303430	496323.333	4439200	8561300	8326900	7109133.33
694	Q16JE9	AAEL013353	FBgn0000308	PFN4	1	7	80.779	0	1213400	173700	86970	491356.667	3648600	2441800	3499900	3196766.67
1154	A0A6I8TH48	AAEL009070	FBgn0261618	LARP1B	2	56	323.31	0	931560	37997	58723	342760	17901000	5905900	8696700	10834533.3
1060	Q16UJ3	AAEL009883	FBgn0287225	ATAD3A	1	20	191.54	0	855470	25238	135010	338572.667	9272800	2650200	10274000	7399000
1126	A0A6I8U795	AAEL025927	FBgn0030918	FBXL13	1	31	323.31	0	900750	55674	32424	329616	9186800	3213600	1492700	4631033.33
1064	A0A6I8TD93	AAEL006582	FBgn0263006	ATP2A1	2	21	285.5	0	804310	52995	126190	327831.667	9301800	6813400	10700000	8938400
1049	A0A6I8TDU5	AAEL006362	FBgn0028646	SLC25A12	4	20	311.16	0	714090	49389	69341	277606.667	7568400	3509700	4761800	5279966.67
1080	Q16SH1	AAEL010585	FBgn0286784	VCP	1	22	323.31	0	779420	12495	18320	270078.333	8723500	1031100	1224600	3659733.33
1006	A0A1S4F5J5	AAEL003726	FBgn0028427	ILK	1	16	323.31	0	589240	75085	54137	239487.333	4677000	5069400	2668700	4138366.67
992	A0A6I8U4P3	AAEL026751	FBgn0003231	SQSTM1	1	15	243.12	0	393420	44121	241110	226217	2581100	2584300	11233000	5466133.33
1112	A0A6I8TEL1	AAEL006876	FBgn0285926	IGF2BP1	1	27	323.31	0	525600	23033	126330	224987.667	3732300	1198400	5033300	3321333.33
1122	Q16TN4	AAEL010159	FBgn0087013	IPO5	1	30	323.31	0	500940	42123	58882	200648.333	5766500	5406200	6346200	5839633.33
1142	A0A6I8T3F0	AAEL000276	FBgn0034641	DCAF1	5	37	323.31	0	24815	213750	335790	191451.667	1096700	18908000	49741000	23248566.7
1089	A0A1S4F1P1	AAEL002407	FBgn0260962	DDB1	1	24	252.58	0	126240	173140	261850	187076.667	2646700	14969000	27505000	15040233.3
1144	A0A1S4FYI5	AAEL013098	FBgn0025725	COPA	2	40	323.31	0	477220	6300.5	39727	174415.833	10339000	618810	2575200	4511003.33
1113	A0A6I8U583	AAEL027249	FBgn0028968	COPG1	1	27	323.31	0	485260	13771	21240	173423.667	7090100	1089000	1509400	3229500
921	J9EB97	AAEL017508	FBgn0027610	SLC25A10	1	12	99.527	0	411630	42157	59926	171237.667	2340800	1681300	1892400	1971500
980	A0A6I8U015	AAEL022091	FBgn0286898	MIA2	3	15	310.48	0	56741	199440	249220	168467	821810	12508000	22413000	11914270
1021	A0A6I8U913	AAEL028182	FBgn0035880	LRP3	1	17	288.44	0	404190	25904	36823	155639	3054300	1131600	1932600	2039500
1138	Q17CT1	AAEL004441	FBgn0037894	IPO9	2	35	323.31	0	135610	70149	135570	113776.333	2184400	6597300	12599000	7126900

526	A0A1S4FKB1	AAEL008740	FBgn0035471	TECR	1	5	67.869	0	205540	52840	55216	104532	1201700	1143000	1116400	1153700
1012	Q176M6	AAEL006315	FBgn0028695	PSMD2	1	16	163.99	0	253000	11677	28875	97850.6667	2724100	1646400	3127100	2499200
1160	Q16EQ1	AAEL015065	FBgn0250789	SPTAN1	4	141	323.31	0	31477	4409.6	196020	77302.2	406340	361800	51586000	17451380
1143	A0A6I8TA52	AAEL004227	FBgn0011225	MYO6	2	39	323.31	0	135290	11693	80723	75902	2327900	1683600	8541100	4184200
1158	Q178J6	AAEL005845	FBgn0250788	SPTBN1	1	111	323.31	0	34676	6930.5	146120	62575.5	1363300	1743300	38543000	13883200
890	Q17NS6	AAEL000622	FBgn0037855	TTC14	1	11	119.36	0	99000	11991	27314	46101.6667	2159900	1442200	1782100	1794733.33
1069	A0A6I8TLG8	AAEL010698	FBgn0042177	IPO4	1	21	273.09	0	55973	24336	25490	35266.3333	1257600	3521000	3107800	2628800
1131	A0A6I8TMQ2	AAEL020992	FBgn0002431	UBR5	6	33	323.31	0	72908	2908.2	12659	29491.7333	3514400	847910	2228100	2196803.33
850	A0A0P6IZN4	AAEL009080	FBgn0026252	MSK	3	11	126.09	0	14891	16092	18107	16363.3333	263300	880580	1052500	732126.667

Supplementary Table 3. ***Ae. aegypti*** **protein interactors in dsRNA knockdown screen.**

ID	UniProt ID	VectorBase ID	FlyBase ID ortholog	HGNC ortholog
1142	A0A6I8T3F0	AAEL000276	FBgn0034641	DCAF1
890	Q17NS6	AAEL000622	FBgn0037855	TTC14
988	A0A6I8T3U9	AAEL001009	FBgn0027598	SH3KBP1
518	A0A1S4EZB3	AAEL001623	FBgn0029133	PSME3
1072	A0A6I8T510	AAEL002178	FBgn0263594	MTHFSD
1089	A0A1S4F1P1	AAEL002407	FBgn0260962	DDB1
789	A0A1S4F345	AAEL002906	FBgn0028692	PSMD1
1012	Q176M6	AAEL006315	FBgn0028695	PSMD2
1049	A0A6I8TDU5	AAEL006362	FBgn0028646	SLC25A12
968	A0A1S4FE42	AAEL006634	FBgn0029969	ACAT1
1112	A0A6I8TEL1	AAEL006876	FBgn0285926	IGF2BP1
1050	Q16YK5	AAEL008517	FBgn0024556	TUFM
1123	Q16Y63	AAEL008658	FBgn0036587	IGFALS
1060	Q16UJ3	AAEL009883	FBgn0287225	ATAD3A
1122	Q16TN4	AAEL010159	FBgn0087013	IPO5
1080	Q16SH1	AAEL010585	FBgn0286784	VCP
437	Q16N14	AAEL012122	FBgn0261396	PSMD3
1085	Q16LC6	AAEL012690	FBgn0037901	EXD2
1023	A0A1S4G5W3	AAEL017116	FBgn0004227	SFPQ
921	J9EB97	AAEL017508	FBgn0027610	SLC25A10
1009	A0A6I8TVN6	AAEL019669	FBgn0022959	YBX1
1059	Q16EU8	AAEL020382	FBgn0012034	ACSS2
1021	A0A6I8U913	AAEL028182	FBgn0035880	LRP3
992	A0A6I8U4P3	AAEL026751	FBgn0003231	SQSTM1

Supplementary Table 4. Primer sequences for cloning.

Target	Primer Name	Sequence (5'-3')*	Reference**
ZIKV C/AC	ZCFor	CGGCGGTTCCCTCGAGATGAAAAACCCAAAAAAGAAATCCG	pCCI-SP6-ZIKV[8]
	ZIKVAC_R	CGGCCTTACTTGTACATCATGCCATAGCTGTGGTCAG	
	ZIKVC_R	CGGCCTTACTTGTACATCACCGTCTCTTCTTCTCCTTCC	
TER94	XhoI_TER94_F	AGGCGGTTCCCTCGAGATGGCCGAAGGAAAGAATGAGG	XM_001654630.2
	TER94_FseI_R	GTCTGCTCGAAGCGGCCGGCCCTAACTGTAGAGATCGTCATCTCCG	

*Underlined are homologous overlaps for In-Fusion cloning.
**mRNA transcripts based on AaegL5.0 (NCBI *Ae. aegypti* annotation release 101)

Supplementary Table 5. Primer sequences for dsRNA synthesis.

Use	Primer Name	Sequence (5'-3')*	Reference**
Knockdown screen	dsAAEL000147_F	**AGTAATACGACTCACTATAGGGAGA**GCGAAATCGGACTAAATTCC	XM_001657642.2
	dsAAEL000147_R	**AGTAATACGACTCACTATAGGGAGA**GCAAACAAGCACTACTCTTC	
	dsAAEL000276_F	**AGTAATACGACTCACTATAGGGAGA**GAAAAGGTCCACAAAACAGG	XM_021852678.1
	dsAAEL000276_R	**AGTAATACGACTCACTATAGGGAGA**TCTGTCTCCTCTTCGAAGAT	
	dsAAEL000622_F	**AGTAATACGACTCACTATAGGGAGA**TTTTCATTGGGCAATCAACC	XM_021845248.1
	dsAAEL000622_R	**AGTAATACGACTCACTATAGGGAGA**TCTTTTACAGTTGCCCTCTC	
	dsAAEL001009_F	**AGTAATACGACTCACTATAGGGAGA**TTGATTGCGTCCAGTAGAAA	XM_021857447.1
	dsAAEL001009_R	**AGTAATACGACTCACTATAGGGAGA**AAGGTACAAAGGGTGTTGTT	
	dsAAEL001623_F	**AGTAATACGACTCACTATAGGGAGA**TCAAGATGTGGATCTCGTTC	XM_001659700.2
	dsAAEL001623_R	**AGTAATACGACTCACTATAGGGAGA**ATAGTCATCAATGTGCGGAT	
	dsAAEL002178_F	**AGTAATACGACTCACTATAGGGAGA**TTGCGCTGAAAAATGTCTTT	XM_021843955.1
	dsAAEL002178_R	**AGTAATACGACTCACTATAGGGAGA**TCTTCGTTCTTTCGGGATTT	
	dsAAEL002407_F	**AGTAATACGACTCACTATAGGGAGA**TTCTAGAATGTGCGGTTCAA	XM_001655181.2
	dsAAEL002407_R	**AGTAATACGACTCACTATAGGGAGA**TTTCGAATTCTCCTCGGTTT	
	dsAAEL002906_F	**AGTAATACGACTCACTATAGGGAGA**CTTGGAGGAAGCTGATTCTT	XM_001662622.2
	dsAAEL002906_R	**AGTAATACGACTCACTATAGGGAGA**GAATGAATCCAATAGCCGTG	
	dsAAEL006315_F	**AGTAATACGACTCACTATAGGGAGA**GGTTTGACTCCGATCGATAA	XM_001651844.2
	dsAAEL006315_R	**AGTAATACGACTCACTATAGGGAGA**TGTTCCGAGCATAGATGAAG	
	dsAAEL006362_F	**AGTAATACGACTCACTATAGGGAGA**AAGTTGCCGAACGAAAAATT	XM_001651912.2
	dsAAEL006362_R	**AGTAATACGACTCACTATAGGGAGA**TGAAAACTCCGCATAGTTGA	
	dsAAEL006634_F	**AGTAATACGACTCACTATAGGGAGA**GAATACGGCCAAGAAAATGG	XM_001657868.2
	dsAAEL006634_R	**AGTAATACGACTCACTATAGGGAGA**AATATGTCAAGTGGGTCACC	
	dsAAEL006876_F	**AGTAATACGACTCACTATAGGGAGA**GCTCGTTGATACTGAGTGAT	XM_021850928.1
	dsAAEL006876_R	**AGTAATACGACTCACTATAGGGAGA**CTACTACAATTCCCGGTGAG	
	dsAAEL008517_F	**AGTAATACGACTCACTATAGGGAGA**GCCATGCCGATTACATTAAG	XM_001659276.2
	dsAAEL008517_R	**AGTAATACGACTCACTATAGGGAGA**TGTCGATTTGATGACCTTGT	
	dsAAEL008658_F	**AGTAATACGACTCACTATAGGGAGA**ACAAATCATGCAAAATGCCA	XM_021855914.1
	dsAAEL008658_R	**AGTAATACGACTCACTATAGGGAGA**CATCACCACTAGAACCAACA	
	dsAAEL009883_F	**AGTAATACGACTCACTATAGGGAGA**CATCCAATCCAGACCTTCAA	XM_001654021.2
	dsAAEL009883_R	**AGTAATACGACTCACTATAGGGAGA**CAGCTTGGAAATTTCTCGAC	
	dsAAEL010159_F	**AGTAATACGACTCACTATAGGGAGA**TTGTGCATATTCGACGATCT	XM_001654246.2
	dsAAEL010159_R	**AGTAATACGACTCACTATAGGGAGA**GTTGGTTTATACACGCTTGG	
	dsAAEL010585_F	**AGTAATACGACTCACTATAGGGAGA**CATTTGCGTTAGACAGCAAT	XM_001654630.2
	dsAAEL010585_R	**AGTAATACGACTCACTATAGGGAGA**GGGGAGAAAAATACGCATTG	
	dsAAEL012122_F	**AGTAATACGACTCACTATAGGGAGA**TTTTCGTGAACCTAGAACGT	XM_001662211.2
	dsAAEL012122_R	**AGTAATACGACTCACTATAGGGAGA**CAGAATAAACCTCGGTTCCT	
	dsAAEL012690_F	**AGTAATACGACTCACTATAGGGAGA**CGCATGATGTCCTACTACTT	XM_001656155.2

	dsAAEL012690_R	**AGTAATACGACTCACTATAGGGAGA**CTCTGGAATCTTACCTGCAC	
	dsAAEL017116_F	**AGTAATACGACTCACTATAGGGAGA**CTGGAGAGCAGTACTTCATC	XM_011494970.2
	dsAAEL017116_R	**AGTAATACGACTCACTATAGGGAGA**CCGTCAAGAAGAAGCATTTC	
	dsAAEL017508_F	**AGTAATACGACTCACTATAGGGAGA**CTTCTACGATCTGGTCAAGG	XM_011495374.2
	dsAAEL017508_R	**AGTAATACGACTCACTATAGGGAGA**AACGATTGAACAGCACTACT	
	dsAAEL019669_F	**AGTAATACGACTCACTATAGGGAGA**CGAGGCTTAATTGCTATCCT	XM_021843784.1
	dsAAEL019669_R	**AGTAATACGACTCACTATAGGGAGA**AGCGCGAAAAGAAAAAGAAA	
	dsAAEL020382_F	**AGTAATACGACTCACTATAGGGAGA**ACATGTGATAACTCCGCTAC	XM_001655469.2
	dsAAEL020382_R	**AGTAATACGACTCACTATAGGGAGA**GCTAAACTCTCCAGGTTTGA	
	dsAAEL028182_F	**AGTAATACGACTCACTATAGGGAGA**AGTTCGAAGATGCCAAGATT	XM_021856314.1
	dsAAEL028182_R	**AGTAATACGACTCACTATAGGGAGA**AATGGCACTCACACATACAT	
	dsAAEL026751_F	**AGTAATACGACTCACTATAGGGAGA**CTTGCAGTGCTTCAACTATG	XM_021844906.1
	dsAAEL026751_R	**AGTAATACGACTCACTATAGGGAGA**TTGGTGTAAAGTTGGGTTCA	
Controls	dsLacZ_F	**TAATACGACTCACTATAGGG**GTCGCCAGCGGCACCGCGCCTTTC	1
	dsLacZ_R	**TAATACGACTCACTATAGGG**CCGGTAGCCAGCGCGGATCATCGG	
	dseGFP_F	**GTAATACGACTCACTATAGGGG**GCGTGCAGTGCTTCAGCCGC	9
	dseGFP_R	**GTAATACGACTCACTATAGGGG**TGGTTGTCGGGCAGCAGCAC	
RNAi reporter assay	dsFLuc_F	**AGTAATACGACTCACTATAGGGAGA**TTGTGGATCTGGATACCGGG	10
	dsFLuc_R	**GTAATACGACTCACTATAGGGAGA**AGCCACCTGATAGCCTTTGT	
*T7 RNA polymerase promoter sequence in bold.<br>**mRNA transcripts based on AaegL5.0 (NCBI *Ae. aegypti* annotation release 101)			

Supplementary Table 6. Primer sequences for RT-qPCR.

Target	Primer Name	Sequence (5'-3')	Reference*
TER94	TER94_F	TGACGCTTATGGATGGTATG	XM_001654630.2
	TER94_R	TACGTCATCAGCCAATTTCA	
Rpn1	Rpn1_F	CGCAGATTGATTCGGCAAGG	XM_001651844.2
	Rpn1_R	ACGTCCCAAAGCAGGATCAG	
AeUBR5	AeUBR5_F	ATGGATCTGCAGGAGATTTC	XM_021850241.1
	AeUBR5_R	AGAAACACTCTTCCGAACAA	
ZIKV	ZIKV_F	GTTGTCGCTGCTGAAATGGA	[11]
	ZIKV_R	GGGGACTCTGATTGGCTGTA	
S7	S7_F	CCAGGCTATCCTGGAGTTG	[11]
	S7_R	GACGTGCTTGCCGGAGAAC	
GAPDH	GAPDH_F	GGTGGTCCAGGGTTTCTTA	[12]
	GAPDH_R	GTTGTCTCCTGCGACTTCA	
*mRNA transcripts based on AaegL5.0 (NCBI *Ae. aegypti* annotation release 101)			

Supplementary Reference

1. Varjak, M. *et al.* Aedes aegypti Piwi4 Is a Noncanonical PIWI Protein Involved in Antiviral Responses. *mSphere* **2**, e00144-17 (2017).

2. Coyaud, E. *et al.* Global interactomics uncovers extensive organellar targeting by Zika Virus. *Mol. Cell. Proteomics* **17**, 2242–2255 (2018).

3. Shah, P. S. *et al.* Comparative Flavivirus-Host Protein Interaction Mapping Reveals Mechanisms of Dengue and Zika Virus Pathogenesis. *Cell* **175**, 1931-1945.e18 (2018).

4. Scaturro, P. *et al.* An orthogonal proteomic survey uncovers novel Zika virus host factors. *Nature* **561**, 253–257 (2018).

5. Savidis, G. *et al.* Identification of Zika Virus and Dengue Virus Dependency Factors using Functional Genomics. *Cell Rep.* **16**, 232–246 (2016).

6. Wang, S. *et al.* Integrin αvβ5 Internalizes Zika Virus during Neural Stem Cells Infection and Provides a Promising Target for Antiviral Therapy. *Cell Rep.* **30**, 969-983.e4 (2020).

7. Li, Y. *et al.* Genome-wide CRISPR screen for Zika virus resistance in human neural cells. *Proc. Natl. Acad. Sci.* **116**, 9527–9532 (2019).

8. Mutso, M. *et al.* Reverse genetic system, genetically stable reporter viruses and packaged subgenomic replicon based on a Brazilian zika virus isolate. *J. Gen. Virol.* **98**, 2712–2724 (2017).

9. McFarlane, M. *et al.* The Aedes aegypti Domino Ortholog p400 Regulates Antiviral Exogenous Small Interfering RNA Pathway Activity and ago-2 Expression. *mSphere* **5**, (2020).

10. Schnettler, E. *et al.* Noncoding Flavivirus RNA Displays RNA Interference Suppressor Activity in Insect and Mammalian Cells. *J. Virol.* **86**, 13486–13500 (2012).

11. Donald, C. L. *et al.* Full Genome Sequence and sfRNA Interferon Antagonist Activity of Zika Virus from Recife, Brazil. *PLoS Negl. Trop. Dis.* **10**, 1–20 (2016).

12. Royle, J. *et al.* Glucose-regulated protein 78 interacts with zika virus envelope protein and contributes to a productive infection. *Viruses* **12**, 524 (2020).
