# Supplementary material for: Analysis of Zika virus capsid-Aedes aegypti mosquito interactome reveals pro-viral host factors critical for establishing infection
Source: Nat Commun. 2021 May 13;12:2766. doi: 10.1038/s41467-021-22966-8 (PMC8119459; doi:10.1038/s41467-021-22966-8)
Supplement: Supplementary file 3 — Reporting Summary [file 41467_2021_22966_MOESM3_ESM.pdf]

nature research

Corresponding author(s): Margus Varjak

Last updated by author(s): Mar 27, 2021

Reporting Summary

Nature Research wishes to improve the reproducibility of the work that we publish. This form provides structure for consistency and transparency in reporting. For further information on Nature Research policies, see our Editorial Policies and the Editorial Policy Checklist.

Statistics

For all statistical analyses, confirm that the following items are present in the figure legend, table legend, main text, or Methods section.

n/a	Confirmed	
☐	☒	The exact sample size (*n*) for each experimental group/condition, given as a discrete number and unit of measurement
☐	☒	A statement on whether measurements were taken from distinct samples or whether the same sample was measured repeatedly
☐	☒	The statistical test(s) used AND whether they are one- or two-sided<br>*Only common tests should be described solely by name; describe more complex techniques in the Methods section.*
☐	☒	A description of all covariates tested
☐	☒	A description of any assumptions or corrections, such as tests of normality and adjustment for multiple comparisons
☐	☒	A full description of the statistical parameters including central tendency (e.g. means) or other basic estimates (e.g. regression coefficient) AND variation (e.g. standard deviation) or associated estimates of uncertainty (e.g. confidence intervals)
☐	☒	For null hypothesis testing, the test statistic (e.g. *F*, *t*, *r*) with confidence intervals, effect sizes, degrees of freedom and *P* value noted<br>*Give P values as exact values whenever suitable.*
☒	☐	For Bayesian analysis, information on the choice of priors and Markov chain Monte Carlo settings
☒	☐	For hierarchical and complex designs, identification of the appropriate level for tests and full reporting of outcomes
☒	☐	Estimates of effect sizes (e.g. Cohen's *d*, Pearson's *r*), indicating how they were calculated

Our web collection on statistics for biologists contains articles on many of the points above.

Software and code

Policy information about availability of computer code

Data collection	1. MaxQuant v.1.6.3.4<br>2. ZEN black 2011 version (with Zeiss LSM 710 confocal microscope)<br>3. Instinct Software v.2.0.1 in GloMax-Multi+ Detection System luminometer<br>4. Image Studio v.1.0.11 with Li-Cor Odyssey CLx<br>5. 7500 software v.2.3 with Applied Biosystems 7500 Fast Real-time PCR<br>6. Image Lab software v.4.1 with Bio-Rad Gel Doc XR+ System
Data analysis	1. Geneious Prime v.2021.0.3<br>2. GraphPad Prism v.7<br>3. Cytoscape v.3.8.2<br>4. MaxQuant v.1.6.3.4<br>5. Andromeda in MaxQuant v.1.6.3.4<br>6. VectorBase with Ae. aegypti AaegL5.3 release<br>7. DAVID v.6.8<br>8. StringDB v.11<br>9. BLAST+ 2.10.1 release<br>10. SAINTq v.0.0.4<br>11. Microsoft Excel v.16.39<br>12. Zeiss ZEN Lite edition v.3.3<br>13. Image Studio Lite v.5.2.5

For manuscripts utilizing custom algorithms or software that are central to the research but not yet described in published literature, software must be made available to editors and reviewers. We strongly encourage code deposition in a community repository (e.g. GitHub). See the Nature Research guidelines for submitting code & software for further information.

Data

Policy information about availability of data

All manuscripts must include a data availability statement. This statement should provide the following information, where applicable:

- Accession codes, unique identifiers, or web links for publicly available datasets
- A list of figures that have associated raw data
- A description of any restrictions on data availability

Mass spectrometry RAW files deposited to the ProteomeXchange Consortium via the PRIDE partner repository with dataset identifier PXD020565 (http://www.ebi.ac.uk/pride/archive/projects/PXD020565). Supplemental data can be accessed through the University of Glasgow Enlighten (http://dx.doi.org/10.5525/gla.researchdata.1020). ZIKV PE243 sequence available from NCBI GenBank (ZIKV/H.sapiens/Brazil/PE243/2015; GenBank: KX197192.1; https://www.ncbi.nlm.nih.gov/nuccore/KX197192.1). Ae. aegypti mRNA sequences from NCBI RefSeq AaegL5.0 (https://www.ncbi.nlm.nih.gov/assembly/GCF_002204515.2) Publicly available data used were obtained from UniProt (Ae. aegypti proteome UP000008820; www.uniprot.org), VectorBase (AaegL5.3; www.vectorbase.org), StringDB (v11.0; www.string-db.org), DAVID (v6.8; https://david.ncifcrf.gov/), OrthoDB (v10.1; www.orthodb.org), FlyBase (FB2020_05; www.flybase.org), HGNC (www.genenames.org), and BioGRID (v4.2; www.thebiogrid.org). Further information and reagent requests including generated unique cell lines and plasmids should be addressed to Alain Kohl, alain.kohl@glasgow.ac.uk. Source data are provided with this paper as Source Data file.

Field-specific reporting

Please select the one below that is the best fit for your research. If you are not sure, read the appropriate sections before making your selection.

☒ Life sciences ☐ Behavioural & social sciences ☐ Ecological, evolutionary & environmental sciences

For a reference copy of the document with all sections, see nature.com/documents/nr-reporting-summary-flat.pdf

Life sciences study design

All studies must disclose on these points even when the disclosure is negative.

Sample size	Sample size was determined according to standards for experimental biology with at least n=3 independent biological repeats with sufficient reproducibility.
Data exclusions	None.
Replication	Experiments were independently repeated at least three times with four technical repeats for specific assay. All attempts at replication were successful and reproducible.
Randomization	Cells taken from the same cultures were randomly assigned to control or experimental conditions for specific assays.
Blinding	Not applicable due to the exploratory nature of the research; experimental design entailed pre-defined conditions/questions.

Reporting for specific materials, systems and methods

We require information from authors about some types of materials, experimental systems and methods used in many studies. Here, indicate whether each material, system or method listed is relevant to your study. If you are not sure if a list item applies to your research, read the appropriate section before selecting a response.

Materials & experimental systems

n/a	Involved in the study	
☐	☒	Antibodies
☐	☒	Eukaryotic cell lines
☒	☐	Palaeontology and archaeology
☒	☐	Animals and other organisms
☒	☐	Human research participants
☒	☐	Clinical data
☒	☐	Dual use research of concern

Methods

n/a	Involved in the study	
☒	☐	ChIP-seq
☒	☐	Flow cytometry
☒	☐	MRI-based neuroimaging

Antibodies

Antibodies used

1. β-Catenin (L54E2) Mouse mAb (IF Preferred) by Cell Signaling Technology (Cat# 2677s)
2. Anti-V5 tag antibody [SV5-Pk1] by Abcam (Cat# ab27671)
3. Anti-beta Catenin antibody by Abcam (Cat# ab16051)
4. Anti-gamma Tubulin antibody by Abcam (Cat# ab11317)
5. Anti-V5 tag antibody by Abcam (Cat# ab9116)

6. Anti-Myc tag antibody [9E10] by Abcam (Cat# ab32)
7. UBR5 monoclonal antibody by Proteintech Group (Cat# 66937-1-Ig) with clone no. 2E7A6
8. VCP polyclonal antibody by Proteintech Group (Cat# 10736-1-AP)
9. p62/SQSTM1 polyclonal antibody by Proteintech Group (Cat# 18420-1-AP)
10. Goat anti-mouse IgG (H+L) cross-adsorbed secondary antibody HRP by Thermo Fisher Scientific (Cat# A16072)
11. Goat anti-mouse IgG (H+L) cross-adsorbed secondary antibody, Alexa Fluor 568 by Thermo Fisher Scientific (Cat# A-11031)
12. Goat anti-mouse IgG (H+L) secondary antibody, DyLight 800 4X PEG by Thermo Fisher Scientific (Cat# SA5-35521)
13. Goat anti-rabbit IgG (H+L) cross-adsorbed secondary antibody , Alexa Fluor 488 by Thermo Fisher Scientific (Cat# A-11008).
14. Goat anti-rabbit IgG (H+L) secondary antibody, DyLight 680 by Thermo Fisher Scientific (Cat# 35568)

Validation

1. Anti-beta Catenin (L54E2) Mouse mAb (IF Preferred) by Cell Signaling Technology (Cat# 2677s). Monoclonal antibody reactive to endogenous levels of human β-Catenin from mice immunized synthetic peptide corresponding to the carboxy terminus of human β-Catenin. Key applications include Western blot, immunofluorescence, and immunoprecipitation. Validated by supplier with species reactivity determined by testing at least one approved application in HeLa and NCl-H28 cells. UniProt ID: P35222. NCBI Gene ID: 1499.
2. Anti-V5 tag antibody [SV5-Pk1] by Abcam (Cat# ab27671). Mouse monoclonal antibody that detects small epitope, termed Pk, present on the P/V proteins of the paramyxovirus, SV5, and produced from mice infected with the paramyxovirus SV5. Validated by supplier and previously by authors in mosquito cells (Varjak et al., 2017. mSphere. https://dx.doi.org/10.1128%2FmSphere.00144-17) during stable or transient expression of V5-tagged recombinant proteins with the sequence epitope from commercially available plasmid, pIB/V5-His (Thermo Fisher Scientific).
3. Anti-beta Catenin antibody by Abcam (Cat# ab16051). Rabbit polyclonal antibody reactive to human beta Catenin suitable for Western blots. Antibody validated by supplier by Western blot of CTNNB1 (β-catenin) knockout HAP1 as well as wild-type HAP1 cell lysates. Produced in rabbits immunized with synthetic peptide corresponding to human beta catenin (C-terminus 750 amino acids).
4. Anti-gamma Tubulin antibody by Abcam (Cat# ab11317). Polyclonal antibody suitable for Western blots and immunofluorescence and reactive to human gamma tubulin. Produced by immunizing rabbits with a synthetic peptide corresponding to the N-terminus (amino acids 35-53) of the human gamma tubulin. Validated by supplier by Western blot in a number of cell lines including A431, HeLa, and NIH3T3.
5. Anti-V5 tag antibody by Abcam (Cat# ab9116). Rabbit polyclonal antibody reactive to V5 tag suitable for Western blots and ELISA. Validated by supplier using ELISA against V5 tag epitope conjugated to BSA.
6. Anti-Myc tag antibody [9E10] by Abcam (Cat# ab32). Monoclonal antibody suitable for Western blot and immunofluorescence and reactive to Myc tag. Produced in mice immunized by a synthetic peptide corresponding to the C-terminus amino acids 400-500 of human Myc tag. Verified by Western blot of supplier using positive control whole cell lysate of recombinant E. coli expressing Myc tag.
7. UBR5 monoclonal antibody by Proteintech Group (Cat# 66937-1-Ig) with clone no. 2E7A6. Anti-human UBR5 mouse monoclonal antibody suitable for Western blot, ELISA, and immunohistochemistry. Validated by supplier through suitable applications in HeLa, MCF-7, and HEK-293 cells and paraffin-embedded tissue. GenBank Accession No.: BC137234. NCBI Gene ID: 51366.
8. VCP polyclonal antibody by Proteintech Group (Cat# 10736-1-AP). Rabbit anti-human VCP polyclonal antibody suitable for Western blot, immunofluorescence, and immunoprecipitation. Validated by suppliers through immunofluorescence of SH-SY5Y cells and Western blot of HeLa cell lysates. GenBank Accession No.: BC007562. NCBI Gene ID: 7415.
9. p62/SQSTM1 polyclonal antibody by Proteintech Group (Cat# 18420-1-AP). Rabbit polyclonal antibody specific to human SQSTM1 suitable for immunofluorescence and Western blots. Validated by Western blot of supplier using lysates of several cell lines including HeLa and HepG2 cells. GenBank Accession No. BC017222. NCBI Gene ID: 8878.
10. Goat anti-mouse IgG (H+L) cross-adsorbed secondary antibody HRP by Thermo Fisher Scientific (Cat# A16072). HRP-conjugated secondary antibody reactive to mouse IgG heavy and light chains suitable for Western blot. Verified by supplier using whole cell lysates of HeLa and Jurkat cells probed with anti-SOD1 mouse monoclonal antibody and chemiluminescent detection of HRP conjugated secondary antibody.
11. Goat anti-mouse IgG (H+L) cross-adsorbed secondary antibody, Alexa Fluor 568 by Thermo Fisher Scientific (Cat# A-11031). Secondary polyclonal antibody conjugated with Alexa Fluor 568 reactive to mouse IgG heavy and light chains. Suitable for immunofluorescence and validated by supplier using HeLa cells stained with anti-alpha tubulin mouse monoclonal antibody.
12. Goat anti-mouse IgG (H+L) secondary antibody, DyLight 800 4X PEG by Thermo Fisher Scientific (Cat# SA5-35521). Secondary polyclonal antibody conjugated with DyLight800 4X PEG reactive to mouse IgG. Supplier verified by Western blot analysis of whole cell extracts of K-562 and U-87 MG probed with anti-SOD1 mouse monoclonal antibody and fluorescent detection using Odyssey Fc imaging system (Li-Cor Biosciences).
13. Goat anti-rabbit IgG (H+L) secondary antibody, DyLight 680 by Thermo Fisher Scientific (Cat# 35568). Secondary polyclonal antibody conjugated with DyLight 680 and reactive to rabbit IgG. Suitable for Western blot with supplier validation using immunofluorescence of alpha tubulin in HeLa cells.

Eukaryotic cell lines

Policy information about cell lines

Cell line source(s)

1. AF5 cells (in-house generated; Varjak et al., 2017. mSphere. https://dx.doi.org/10.1128%2FmSphere.00144-17); available from ECACC, Cat# 19022601)
2. A549 cells (available from ECACC, Cat# 86012804)
3. A549-Npro cells (a kind gift from RE Randall, University of St. Andrews; refer to Hilton et al., 2006 https://dx.doi.org/10.1128%2FJVI.01145-06)

Authentication

1. AF5 cell line [Aag2-AF5] commercially available with authentication from ECACC; A single cell clone derived from Aag2 cell line; immunocompetent and frequently tested for functional RNA interference using in-house generated RNAi sensor assay (refer to Varjak et al., 2017 https://dx.doi.org/10.1128%2FmSphere.00144-17 and Fredericks et al., 2019 PLoS Negl Trop Dis. https://doi.org/10.1371/journal.pntd.0007346).
Editor:); assessed by qPCR for S7 housekeeping gene using specific primers.
2. A549 and A549-NPro were not authenticated specifically.

Mycoplasma contamination	Cell lines tested negative for mycoplasma.
Commonly misidentified lines<br>(See ICLAC register)	None.
